# Phase Angle Reference Values Measured Using InBody770 in Japanese Individuals With Normal Fluid Balance

**DOI:** 10.7759/cureus.111766

**Published:** 2026-06-29

**Authors:** Naoto Kisaichi, Kentaro Kawai, Jiro Kurakata, Kei Suzuki

**Affiliations:** 1 Department of Rehabilitation, The Institute of Neurosurgery and Rehabilitation, Suzuki Kei Yasuragi Clinic, Tachikawa, JPN; 2 Division of Physical Therapy, Department of Rehabilitation, Faculty of Health Sciences, University of Tokyo Health Sciences, Tama, JPN; 3 Department of Neurosurgery, The Institute of Neurosurgery and Rehabilitation, Suzuki Kei Yasuragi Clinic, Tachikawa, JPN

**Keywords:** bioelectrical impedance analysis, ecw/tbw, inbody770, phase angle, standardized phase angle

## Abstract

Introduction

This survey aimed to present reference values for phase angle (PhA) measured using the InBody770 (InBody Japan Inc., Koto City, Tokyo, Japan) in Japanese individuals with normal fluid balance, defined as an extracellular water/total body water (ECW/TBW) ratio <0.4.

Materials and methods

This retrospective survey analyzed data obtained from 15,999 measurements of Japanese individuals aged eight to 99 years who were assessed using the InBody770 body composition analyzer. Exclusion criteria were unsuccessful InBody measurements due to impedance errors, individuals with multiple measurement records, aged <10 years, those with a body mass index (BMI) ≥40.0 kg/m², and those with an ECW/TBW ratio ≥0.4. The survey included age, sex, height, weight, BMI, skeletal muscle index (SMI), ECW/TBW, and whole-body PhA.

Results

After applying the exclusion criteria, the final analysis included 2,562 datasets (959 men and 1,603 women). PhA reference values stratified by age, sex, and BMI using the InBody770 were established. PhA generally decreased with advancing age and tended to be higher in individuals with higher BMI and in men. The overall mean PhA was 5.4 ± 0.8° in men and 4.7 ± 0.5° in women.

Conclusions

The PhA reference values established in this survey using the InBody770 may serve as a basis for advancing research on PhA and for calculating standardized PhA (SPA) values in Asian populations.

## Introduction

Phase angle (PhA), an indicator directly calculated from measurements obtained by bioelectrical impedance analysis (BIA) without using predictive equations [1], reflects the physiological functional level of cells, with higher values indicating higher cell density, membrane integrity, and cellular function [2,3]. PhA is associated with body fat percentage [4], age, sex, body mass index (BMI) [4-6], population characteristics [4,5,7], body composition [7], extracellular water/total body water (ECW/TBW) [8], malignant effusion [9], nutrition [9,10], inflammation [10], muscle strength [11], muscle quality [12], falls [13], handgrip strength [14], sarcopenia [9,15], and physical activity level [16].

However, because PhA varies according to age, sex, and BMI [4-6], the importance of the standardized PhA (SPA), which provides a Z-score for PhA, has been reported [17]. SPA is calculated using the mean and standard deviation of PhA obtained from a healthy population stratified by age, sex, and BMI: SPA = (observed PhA − mean PhA)/standard deviation of PhA [18]. By comparing a patient’s results with the mean values of a specific population, the SPA can enhance its ability to predict clinical outcomes [2], and its usefulness has been demonstrated in kidney transplant recipients [19], older adults [20], and patients with cancer [14]. Additionally, the SPA is useful for predicting the survival of patients with COVID-19 [21]. Therefore, SPA enables comparisons across different clinical settings and studies.

However, despite the many reported associations with PhA, SPA has not been widely used in fields besides nutrition science. Research on PhA and sarcopenia is one example. The diagnostic criteria for sarcopenia differ among populations, such as those proposed by the European Working Group on Sarcopenia in Older People 2 and the Asian Working Group for Sarcopenia 2025 [22,23]. Research using SPA for diseases with population-specific diagnostic criteria, such as sarcopenia, suggests that PhA reference values adjusted for age, sex, and BMI according to population characteristics are necessary. Currently, the only available PhA reference values stratified by age, sex, and BMI are based on a German database of 230,337 individuals aged six to 102 years [4]. Other reports included data from 5,225 Western Europeans aged 15-94 years [24] and 1,967 individuals from diverse populations aged 18-94 years [5]; however, these studies did not consider BMI. To the best of our knowledge, no studies have reported PhA reference values in Asian populations stratified simultaneously by age, sex, and BMI.

Although PhA reference values required for calculating SPA should be based on a “healthy population,” the specific criteria for “health” have not been defined. The 1948 Constitution of the World Health Organization (WHO) defined health as “a state of complete physical, mental, and social well-being and not merely the absence of disease or infirmity” [25]. It is difficult to evaluate whether individuals meet these criteria or establish a baseline health level in a target population. In a previous study providing PhA reference values [4], participants with no renal, endocrine, or cardiac diseases that could affect fluid balance were included. However, this was determined using subjective questionnaires, and no objective indicators of actual fluid balance were reported. ECW/TBW is widely used as an indicator of fluid balance, and an ECW/TBW ratio ≥0.4 increases the likelihood of edema and may affect BIA measurements [26-28]. This survey evaluated ECW/TBW and presented PhA reference values for Japanese individuals with normal fluid balance, defined as an ECW/TBW ratio <0.4. However, ECW/TBW <0.4 should not be interpreted as indicating a healthy population. Rather, it was used in this survey as an objective indicator of normal fluid balance. Therefore, this survey aimed to present PhA reference values measured using the InBody770 in Japanese individuals with normal fluid balance, defined as an ECW/TBW ratio <0.4.

## Materials and methods

This was a retrospective survey. The inclusion criteria were as follows: Japanese individuals aged eight to 99 years who underwent measurements using the InBody770 (InBody Japan Inc., Koto City, Tokyo, Japan) at The Institute of Neurosurgery and Rehabilitation, Suzuki Kei Yasuragi Clinic between December 2010 and September 2025. The study population consisted of individuals attending a medical institution. Because this survey was based on a retrospective database, all available measurements obtained during the study period were initially extracted. A total of 15,999 individuals met these criteria and were included in the survey. Each dataset was anonymized before analysis by assigning random, non-sequential numerical codes to prevent the identification of individuals.

The exclusion criteria were as follows: cases in which InBody measurements were not properly performed according to impedance criteria, individuals with multiple InBody measurement histories, individuals aged <10 years, individuals with BMI ≥40.0 kg/m², and cases with an ECW/TBW ratio ≥0.4. The criteria for determining improper measurements were as follows: any site in which impedance at 5-500 kHz showed even a slight reversal, impedance >50 Ω for the trunk or 700 Ω for the limbs at any frequency, or a sudden drop of ≥10 Ω in the trunk or ≥100 Ω in the limbs across the frequency range of 1-1,000 kHz. Individuals aged <10 years were excluded because the sample size in this age group was insufficient for age-, sex-, and BMI-stratified analyses. Individuals with BMI ≥40.0 kg/m² were excluded because increased tissue fluid content or pathological fluid overload may lead to a reported inverse correlation with PhA [4]. The ECW/TBW exclusion threshold of ≥0.4 was adopted based on previous studies and InBody criteria, as this cutoff is widely used to suggest the presence of edema or fluid overload and may influence body composition measurements [26-28].

Measurements were performed by physical therapists, occupational therapists, and athletic trainers with sufficient experience. Before measurement, the participants were instructed to ensure that at least two hours had passed since their last meal, undergo measurement before exercise, and void their bladders. To avoid measurements immediately following prolonged sitting or supine positioning, the participants stood for approximately five minutes before testing. The measurement posture was barefoot standing with both heels placed on the electrode plates and both upper limbs abducted slightly so that the fingers grasped the hand electrodes, ensuring contact with the designated electrode areas. The examiners confirmed that the arms did not touch the trunk, the elbows were fully extended, and the inner thighs did not make contact. The participants were instructed to refrain from movement and conversation during the measurement.

Statistical analysis

The survey included age, sex, height, weight, BMI, skeletal muscle index (SMI), ECW/TBW, and whole-body PhA. No missing data were present in the variables used for analysis. SPA calculation requires the mean and standard deviation of PhA stratified by age, sex, and BMI. Therefore, after excluding data based on the aforementioned criteria, the initial InBody measurements for each participant were stratified by sex, age (10-19, 20-29, 30-39, 40-49, 50-59, 60-69, 70-79, 80-89, and 90-99 years), and BMI (<18.5, 18.5-24.9, 25.0-29.9, and 30.0-39.9 kg/m²). The mean, standard deviation, and percentiles (5th, 25th, 50th, 75th, and 95th) of PhA were calculated for each stratum. Normality of PhA in all strata was assessed using the Shapiro-Wilk test. Statistical significance was set at p < 0.05. All statistical analyses were performed using Modified R Commander version 2.9-1 for Windows.

## Results

A total of 15,999 Japanese individuals (6,338 men and 9,661 women) were included in the survey. The demographic characteristics of the survey population are shown in Table 1. Men had higher mean values for height, weight, BMI, SMI, and PhA than women. Women had a higher mean age and ECW/TBW values than men.

**Table 1:** Demographic characteristics of this survey population BMI: body mass index; SMI: skeletal muscle index; ECW/TBW: extracellular water/total body water; PhA: phase angle Values are presented as mean ± standard deviation

	Men (N = 6,338)	Women (N = 9,661)	Total (N = 15,999)
Age (years)	67.1 ± 16.1	69.8 ± 13.2	68.7 ± 14.5
Height (cm)	166.2 ± 6.5	152.5 ± 6.4	157.9 ± 9.3
Weight (kg)	70.3 ± 14.3	57.6 ± 12.1	62.6 ± 14.4
BMI (kg/m^2^)	25.3 ± 4.3	24.7 ± 4.7	25.0 ± 4.6
SMI (kg/m^2^)	7.6 ± 1.0	6.1 ± 0.8	6.7 ± 1.1
ECW/TBW	0.388 ± 0.010	0.393 ± 0.008	0.391 ± 0.010
PhA (°)	5.2 ± 0.9	4.5 ± 0.6	4.8 ± 0.8

**Figure 1:**
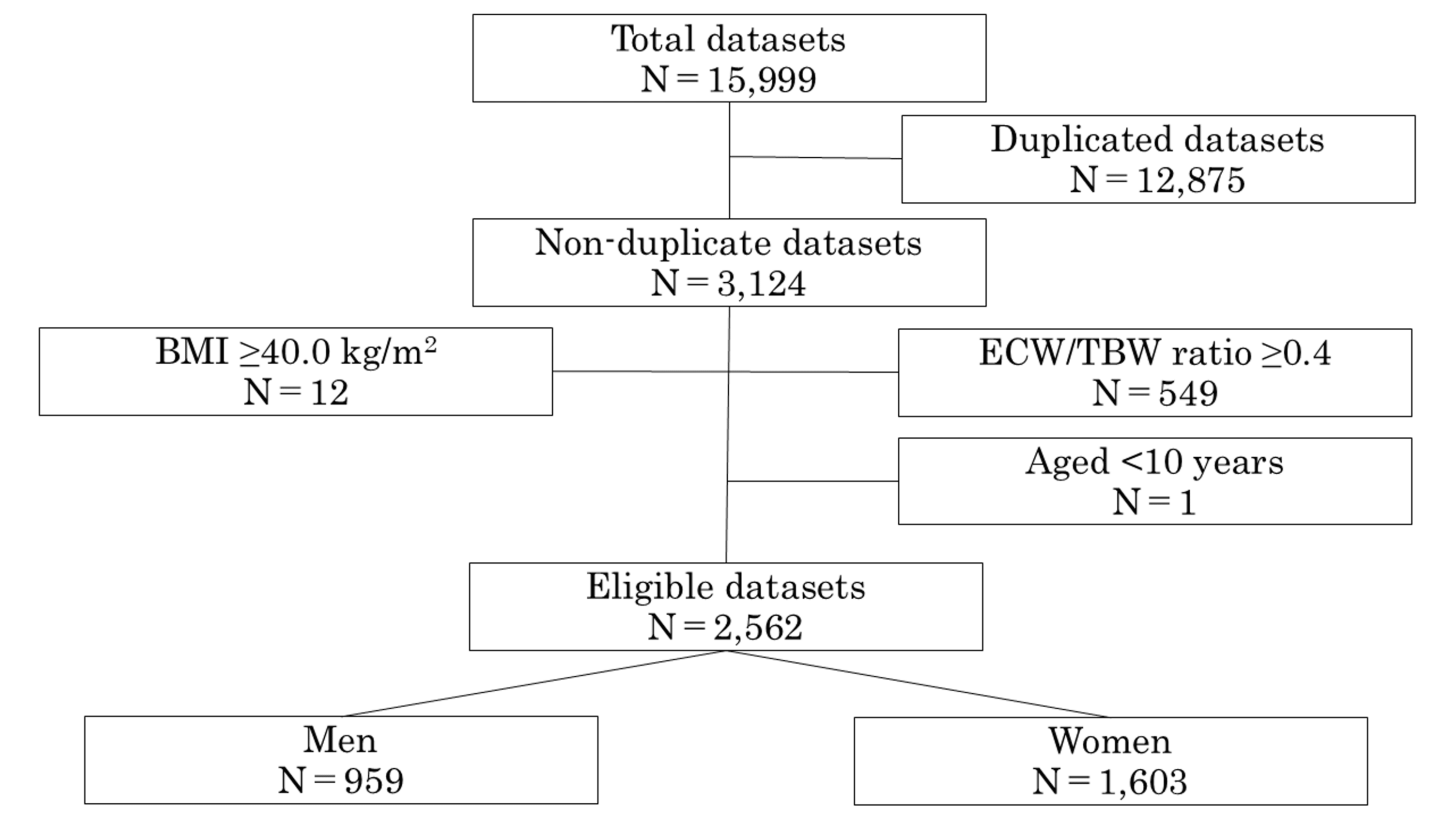
Flowchart of participant selection

After applying the exclusion criteria, 2,562 datasets were included in the final analysis, comprising 959 men (mean age 63.3 ± 16.6 years) and 1,603 women (mean age 66.4 ± 14.7 years). A flowchart of participant selection is shown in Figure 1. Of the 15,999 datasets initially identified, 12,875 duplicate datasets were excluded. Among the remaining 3,124 nonduplicated datasets, 12 datasets with a BMI ≥40.0 kg/m², 549 datasets with an ECW/TBW ratio ≥0.4, and one dataset from an individual aged <10 years were excluded, resulting in 2,562 datasets for the final analysis. Among these exclusion criteria, an ECW/TBW ratio ≥0.4 was the most common reason for exclusion.

The results of the Shapiro-Wilk test performed on PhA in all strata are shown in Tables 2-3. Some strata did not meet the assumption of normality in either sex.

**Table 2:** Results of normality testing for PhA stratified by age and BMI in men BMI: body mass index; W: Shapiro-Wilk test statistic; N/A: insufficient sample size for analysis Normality assessed using the Shapiro-Wilk test. ^*^p<0.05

	BMI <18.5kg/m^2^ (N = 29)	BMI 18.5-24.9kg/m^2^ (N = 487)	BMI 25.0-29.9kg/m^2^ (N = 339)	BMI 30.0-39.9kg/m^2^ (N = 104)
Age (10-19 years)	N = 1	N = 9	N = 2	N = 1
W	N/A	0.886	N/A	N/A
Age (20-29 years)	N = 3	N = 24	N = 6	N = 5
W	N/A	0.955	0.905	0.853
Age (30-39 years)	N = 1	N = 17	N = 15	N = 11
W	N/A	0.896	0.973	0.876
Age (40-49 years)	N = 0	N = 30	N = 43	N = 30
W	N/A	0.973	0.950	0.938
Age (50-59 years)	N = 3	N = 46	N = 56	N = 22
W	N/A	0.982	0.992	0.950
Age (60-69 years)	N = 5	N = 106	N = 71	N = 16
W	0.854	0.970^*^	0.965^*^	0.979
Age (70-79 years)	N = 10	N = 168	N = 114	N = 13
W	0.927	0.982^*^	0.983	0.826^*^
Age (80-89 years)	N = 6	N = 83	N = 30	N = 5
W	0.922	0.979	0.956	0.833
Age (90-99 years)	N = 0	N = 4	N = 2	N = 1
W	N/A	0.797	N/A	N/A

**Table 3:** Results of normality testing for PhA stratified by age and BMI in women PhA: phase angle; BMI: body mass index; W: Shapiro-Wilk test statistic; N/A: insufficient sample size for analysis Normality assessed using the Shapiro-Wilk test. ^*^p < 0.05;  ^**^p < 0.01

	BMI <18.5kg/m^2^ (N = 131)	BMI 18.5-24.9kg/m^2^ (N = 915)	BMI 25.0-29.9kg/m^2^ (N = 425)	BMI 30.0-39.9kg/m^2^ (N = 132)
Age (10-19 years)	N = 1	N = 14	N = 4	N = 0
W	N/A	0.967	0.750^**^	N/A
Age (20-29 years)	N = 5	N = 29	N = 2	N = 2
W	0.793	0.965	N/A	N/A
Age (30-39 years)	N = 11	N = 26	N = 5	N = 5
W	0.878	0.971	0.859	0.789
Age (40-49 years)	N = 6	N = 50	N = 28	N = 20
W	0.914	0.980	0.970	0.961
Age (50-59 years)	N = 12	N = 77	N = 52	N = 41
W	0.977	0.987	0.981	0.978
Age (60-69 years)	N = 29	N = 189	N = 116	N = 39
W	0.959	0.986	0.989	0.981
Age (70-79 years)	N = 45	N = 380	N = 168	N = 21
W	0.951	0.989^**^	0.980^*^	0.842^**^
Age (80-89 years)	N = 22	N = 148	N = 47	N = 4
W	0.933	0.952^**^	0.986	0.893
Age (90-99 years)	N = 0	N = 2	N = 3	N = 0
W	N/A	N/A	N/A	N/A

The age-, sex-, and BMI-specific reference values and percentile distributions of PhA are presented in Tables 4-5. In both sexes, PhA generally decreased with advancing age. Across most age groups, individuals in higher BMI categories tended to exhibit higher PhA values than those in lower BMI categories. Furthermore, men generally had higher PhA values than women within corresponding age and BMI strata.

**Table 4:** Results of the datasets stratified by age and BMI in men SD: standard deviation; BMI: body mass index; SMI: skeletal muscle index; ECW/TBW: extracellular water/total body water; PhA: phase angle; N/A: insufficient sample size for analysis Percentile values were calculated from the distribution of observations within each age- and BMI-specific stratum

	BMI <18.5kg/m^2^ (N = 29)							BMI 18.5-24.9kg/m^2^ (N = 487)							BMI 25.0-29.9kg/m^2^ (N = 399)							BMI 30.0-39.9kg/m^2^ (N = 104)						
	Mean	SD	Percentile					Mean	SD	Percentile					Mean	SD	Percentile					Mean	SD	Percentile				
Age (10-19 years)	N = 1		5th	25th	50th	75th	95th	N = 9		5th	25th	50th	75th	95th	N = 2		5th	25th	50th	75th	95th	N = 1		5th	25th	50th	75th	95th
Age (years)	15.3	N/A	15.3	15.3	15.3	15.3	15.3	15.6	2.6	11.5	14.2	15.8	17.3	19.0	17.2	1.9	15.5	16.2	17.2	18.1	18.8	15.6	N/A	15.6	15.6	15.6	15.6	15.6
Height (cm)	171.0	N/A	171.0	171.0	171.0	171.0	171.0	170.8	12.2	151.4	165.1	170.5	181.4	186.4	174.1	5.4	169.2	171.4	174.1	176.8	179.0	162.6	N/A	162.6	162.6	162.6	162.6	162.6
Weight (kg)	51.7	N/A	51.7	51.7	51.7	51.7	51.7	63.6	11.1	48.4	55.1	62.9	74.4	78.5	86.6	5.9	81.3	83.6	86.6	89.5	91.8	91.9	N/A	91.9	91.9	91.9	91.9	91.9
BMI (kg/m^2^)	17.7	N/A	17.7	17.7	17.7	17.7	17.7	21.7	1.8	18.6	20.5	22.1	22.9	23.8	28.6	0.2	28.4	28.5	28.6	28.6	28.7	34.8	N/A	34.8	34.8	34.8	34.8	34.8
SMI (kg/m^2^)	6.6	N/A	6.6	6.6	6.6	6.6	6.6	7.6	1.1	6.2	6.5	7.7	8.5	9.1	8.4	0.2	8.3	8.4	8.4	8.5	8.6	9.2	N/A	9.2	9.2	9.2	9.2	9.2
ECW/TBW	0.387	N/A	0.387	0.387	0.387	0.387	0.387	0.377	0.009	0.366	0.371	0.376	0.382	0.393	0.372	0.008	0.365	0.368	0.372	0.375	0.378	0.372	N/A	0.372	0.372	0.372	0.372	0.372
PhA (°)	5.0	N/A	5.0	5.0	5.0	5.0	5.0	5.8	0.9	4.3	5.2	6.1	6.5	6.9	6.7	0.9	5.8	6.2	6.7	7.1	7.5	6.8	N/A	6.8	6.8	6.8	6.8	6.8
Age (20-29 years)	N = 3							N = 24							N = 6							N = 5						
Age (years)	24.3	3.4	21.2	22.0	23.0	26.0	28.4	24.0	2.8	21.0	21.0	23.5	27.0	28.0	25.7	2.5	21.8	24.5	26.5	27.8	28.0	25.0	3.0	21.2	22.0	26.0	27.0	28.6
Height (cm)	167.2	9.1	160.2	160.8	161.5	170.8	178.2	173.2	5.6	164.2	170.5	173.5	175.0	184.0	169.0	5.1	161.0	170.1	170.5	170.9	173.1	172.7	5.8	165.8	169.0	172.3	175.2	180.6
Weight (kg)	49.3	6.6	44.3	44.6	45.0	51.8	57.2	62.8	6.2	53.3	58.6	61.4	67.9	72.3	77.9	5.2	71.0	74.2	77.8	82.4	84.3	107.6	5.3	100.9	101.8	110.1	112.7	112.8
BMI (kg/m^2^)	17.6	0.4	17.3	17.3	17.3	17.7	18.0	20.9	1.5	18.8	19.9	20.7	22.0	23.2	27.3	1.3	25.6	26.3	27.3	28.5	28.8	36.1	1.1	34.4	35.6	36.7	37.0	37.1
SMI (kg/m^2^)	6.4	0.7	5.9	5.9	6.0	6.7	7.3	7.8	0.6	6.9	7.4	7.8	8.1	8.8	8.3	0.4	7.9	8.0	8.2	8.7	8.8	9.2	0.3	8.9	9.1	9.2	9.3	9.7
ECW/TBW	0.383	0.002	0.380	0.381	0.382	0.384	0.386	0.374	0.005	0.367	0.371	0.376	0.378	0.380	0.374	0.004	0.369	0.373	0.374	0.377	0.380	0.375	0.001	0.374	0.374	0.375	0.377	0.377
PhA (°)	5.0	0.3	4.6	4.9	5.2	5.2	5.2	6.3	0.4	5.6	6.0	6.4	6.7	6.9	6.3	0.4	5.9	6.0	6.3	6.5	7.0	6.5	0.3	6.2	6.3	6.3	6.6	6.9
Age (30-39 years)	N = 1							N = 17							N = 15							N = 11						
Age (years)	38.0	N/A	38.0	38.0	38.0	38.0	38.0	34.6	3.5	30.0	30.0	35.0	38.0	39.0	36.1	2.7	30.7	34.5	37.0	38.0	39.0	34.2	2.6	30.5	32.0	34.0	36.0	38.0
Height (cm)	179.0	N/A	179.0	179.0	179.0	179.0	179.0	170.7	4.0	165.2	168.0	170.0	174.0	177.2	170.7	6.7	163.9	165.2	169.5	174.8	183.3	170.7	4.4	166.0	166.9	169.0	174.4	177.5
Weight (kg)	56.2	N/A	56.2	56.2	56.2	56.2	56.2	62.1	4.2	56.3	59.1	62.3	64.7	68.1	79.7	6.0	71.4	76.3	78.6	82.0	90.1	94.7	7.5	84.4	91.5	93.6	97.0	108.0
BMI (kg/m^2^)	17.5	N/A	17.5	17.5	17.5	17.5	17.5	21.3	1.1	19.3	20.6	21.3	22.1	22.9	27.4	1.4	25.3	26.4	27.2	27.9	29.7	32.5	2.0	30.1	30.7	32.1	34.3	35.4
SMI (kg/m^2^)	6.9	N/A	6.9	6.9	6.9	6.9	6.9	7.3	0.5	6.7	7.2	7.4	7.6	7.9	8.2	0.5	7.5	7.9	8.2	8.5	8.9	9.0	0.6	8.1	8.7	8.9	9.3	9.9
ECW/TBW	0.383	N/A	0.383	0.383	0.383	0.383	0.383	0.376	0.007	0.366	0.372	0.375	0.380	0.386	0.375	0.006	0.367	0.371	0.375	0.377	0.384	0.373	0.008	0.363	0.366	0.375	0.379	0.383
PhA (°)	5.2	N/A	5.2	5.2	5.2	5.2	5.2	5.9	0.7	4.5	5.6	5.9	6.4	6.7	6.3	0.6	5.5	6.0	6.1	6.6	7.2	6.5	0.7	5.8	6.0	6.2	7.2	7.6
Age (40-49 years)	N = 0							N = 30							N = 43							N = 30						
Age (years)	N/A	N/A	N/A	N/A	N/A	N/A	N/A	45.1	2.8	41.0	43.0	45.0	48.0	49.0	45.1	2.9	40.1	42.5	46.0	48.0	49.0	45.0	2.4	41.5	43.3	45.0	46.8	49.0
Height (cm)	N/A	N/A	N/A	N/A	N/A	N/A	N/A	169.4	5.5	163.6	165.9	168.5	173.9	178.1	171.1	6.9	160.0	166.0	170.4	177.1	181.8	171.3	5.6	165.1	167.2	170.0	174.6	180.3
Weight (kg)	N/A	N/A	N/A	N/A	N/A	N/A	N/A	63.4	6.8	51.4	58.9	64.8	67.1	75.1	81.1	8.8	67.9	73.8	80.2	87.7	93.0	97.7	10.2	84.5	89.5	96.5	104.9	114.5
BMI (kg/m^2^)	N/A	N/A	N/A	N/A	N/A	N/A	N/A	22.0	1.7	19.6	20.6	22.1	23.2	24.7	27.6	1.6	25.1	26.3	27.7	28.8	29.8	33.2	2.3	30.6	31.1	33.2	34.7	36.8
SMI (kg/m^2^)	N/A	N/A	N/A	N/A	N/A	N/A	N/A	7.5	0.5	6.6	7.1	7.6	8.0	8.2	8.3	0.6	7.4	8.0	8.3	8.6	9.4	9.1	0.5	8.3	8.7	9.1	9.4	9.9
ECW/TBW	N/A	N/A	N/A	N/A	N/A	N/A	N/A	0.381	0.006	0.370	0.376	0.381	0.385	0.390	0.376	0.006	0.369	0.373	0.375	0.380	0.386	0.380	0.007	0.369	0.373	0.380	0.386	0.389
PhA (°)	N/A	N/A	N/A	N/A	N/A	N/A	N/A	5.7	0.6	4.8	5.4	5.7	6.2	6.7	6.2	0.5	5.3	5.9	6.4	6.6	6.9	6.1	0.5	5.3	5.7	6.0	6.6	7.0
Age (50-59 years)	N = 3							N = 46							N = 56							N = 22						
Age (years)	56.7	0.5	56.1	56.5	57.0	57.0	57.0	54.4	2.7	51.0	52.3	54.5	56.0	59.0	54.7	3.0	50.0	52.0	55.0	57.3	59.0	53.9	2.8	50.0	52.0	53.0	56.8	58.0
Height (cm)	170.4	2.0	168.2	169.2	170.3	171.7	172.7	170.3	6.6	160.4	165.4	169.8	172.8	183.7	171.3	5.7	161.9	168.3	171.5	174.5	180.1	172.0	5.3	165.9	167.8	171.8	174.0	183.2
Weight (kg)	51.7	1.1	50.4	51.2	52.1	52.5	52.8	67.0	8.3	54.9	60.7	66.0	72.1	81.9	79.7	6.8	70.9	74.7	77.9	84.8	92.4	97.0	8.3	85.0	89.5	96.3	104.5	109.7
BMI (kg/m^2^)	17.8	0.1	17.7	17.8	17.8	17.9	18.0	23.0	1.7	20.1	22.4	23.6	24.3	24.8	27.1	1.4	25.1	26.0	26.8	28.4	29.4	32.8	2.1	30.4	30.9	32.6	33.9	36.1
SMI (kg/m^2^)	6.7	0.3	6.3	6.5	6.7	6.9	7.1	7.5	0.6	6.5	7.1	7.6	7.9	8.3	8.2	0.5	7.6	7.9	8.1	8.6	9.0	9.2	0.6	8.2	8.8	9.2	9.7	10.1
ECW/TBW	0.381	0.002	0.378	0.380	0.381	0.382	0.383	0.381	0.007	0.371	0.375	0.381	0.386	0.395	0.380	0.005	0.372	0.375	0.380	0.383	0.389	0.384	0.005	0.375	0.382	0.384	0.388	0.393
PhA (°)	5.4	0.5	4.8	5.2	5.7	5.7	5.7	5.6	0.6	4.6	5.2	5.5	6.1	6.6	5.9	0.5	5.0	5.5	5.8	6.1	6.6	5.8	0.5	5.0	5.6	5.8	6.0	6.6
Age (60-69 years)	N = 5							N = 106							N = 71							N = 16						
Age (years)	65.6	2.1	62.6	65.0	66.0	67.0	67.8	65.0	2.6	61.0	63.0	65.0	67.0	69.0	64.7	2.7	60.0	63.0	65.0	67.0	69.0	64.4	2.8	60.8	61.8	64.0	66.3	69.0
Height (cm)	166.0	5.5	160.0	160.1	165.9	170.0	173.2	167.2	5.7	158.2	163.0	167.0	171.2	177.4	168.2	5.4	159.5	164.5	167.5	172.4	177.1	168.7	5.1	160.6	166.0	168.1	170.8	177.4
Weight (kg)	49.3	2.4	46.4	46.8	49.7	51.6	52.2	63.2	5.8	52.6	59.8	62.5	67.4	72.4	76.6	6.8	67.6	71.5	75.8	81.0	88.6	91.4	8.6	80.8	85.4	89.0	98.9	105.3
BMI (kg/m^2^)	17.9	0.3	17.4	17.9	18.1	18.1	18.3	22.6	1.6	19.4	21.7	22.9	23.8	24.7	27.0	1.5	25.2	25.7	26.8	28.3	29.7	32.1	1.8	30.1	30.7	31.6	32.7	35.4
SMI (kg/m^2^)	6.3	0.5	5.8	6.0	6.1	6.7	7.0	7.3	0.5	6.3	7.0	7.3	7.6	8.1	8.1	0.6	7.2	7.7	8.1	8.5	9.0	8.8	0.7	7.5	8.2	9.0	9.2	9.8
ECW/TBW	0.393	0.005	0.387	0.390	0.392	0.397	0.399	0.385	0.006	0.376	0.381	0.385	0.390	0.394	0.383	0.006	0.374	0.378	0.382	0.387	0.395	0.388	0.008	0.376	0.383	0.388	0.395	0.398
PhA (°)	4.4	0.3	4.1	4.2	4.3	4.7	4.8	5.3	0.6	4.4	4.8	5.4	5.7	6.2	5.6	0.6	4.8	5.3	5.7	6.0	6.4	5.4	0.6	4.5	5.1	5.4	5.8	6.3
Age (70-79 years)	N = 10							N = 168							N = 114							N = 13						
Age (years)	75.4	2.8	70.9	74.0	75.5	77.8	79.0	74.1	2.7	70.0	72.0	74.0	76.0	79.0	74.5	2.9	70.0	72.0	74.5	77.0	79.0	74.5	2.7	70.6	72.0	75.0	76.0	79.0
Height (cm)	165.5	8.5	154.0	158.2	165.0	172.6	177.5	164.5	6.0	155.0	160.4	164.1	168.4	174.1	164.4	5.9	154.8	160.6	164.8	167.9	174.1	164.5	5.9	156.4	160.0	164.3	169.8	172.7
Weight (kg)	47.4	6.0	39.6	42.4	47.4	50.4	56.7	61.1	6.5	50.9	56.9	61.3	65.4	72.5	73.1	6.2	63.5	68.1	73.3	77.2	82.7	84.7	6.0	74.7	79.5	85.8	89.1	92.1
BMI (kg/m^2^)	17.2	1.0	15.6	16.8	17.5	18.1	18.2	22.6	1.6	19.5	21.5	22.9	23.7	24.8	27.0	1.3	25.3	26.0	26.9	27.8	29.4	31.3	1.0	30.2	30.3	31.2	31.8	33.0
SMI (kg/m^2^)	6.1	0.8	5.1	5.4	6.1	6.7	7.3	7.1	0.5	6.2	6.7	7.1	7.4	8.0	7.8	0.5	6.9	7.4	7.8	8.2	8.6	8.4	0.7	7.3	7.5	8.6	8.8	9.3
ECW/TBW	0.392	0.005	0.384	0.389	0.393	0.396	0.398	0.388	0.006	0.378	0.384	0.389	0.392	0.396	0.388	0.005	0.380	0.384	0.388	0.392	0.397	0.390	0.006	0.381	0.386	0.391	0.393	0.398
PhA (°)	4.5	0.6	3.9	4.2	4.4	4.7	5.5	5.0	0.5	4.2	4.6	5.0	5.3	5.9	5.2	0.5	4.4	4.8	5.1	5.5	6.0	5.2	0.5	4.7	4.9	5.0	5.3	6.1
Age (80-89 years)	N = 6							N = 83							N = 30							N = 5						
Age (years)	82.3	2.4	80.0	80.3	81.5	84.3	85.8	82.8	2.4	80.0	81.0	82.0	84.0	87.0	83.0	2.6	80.0	81.0	82.0	84.8	87.6	82.2	1.3	80.4	82.0	82.0	83.0	83.8
Height (cm)	165.5	7.0	158.4	158.6	165.0	172.4	173.3	161.6	5.5	153.3	157.5	162.0	165.0	170.9	160.0	6.4	150.9	156.3	159.0	162.9	173.2	163.9	8.9	150.8	160.0	166.4	171.5	172.7
Weight (kg)	48.2	4.1	44.0	44.8	47.3	50.4	54.4	58.9	6.1	49.5	54.9	58.4	64.2	68.3	68.7	6.4	60.0	64.4	67.6	71.2	81.8	83.1	8.8	69.7	79.5	88.6	90.0	90.1
BMI (kg/m^2^)	17.6	0.7	16.5	17.4	17.6	18.2	18.4	22.5	1.6	19.7	21.4	22.8	24.0	24.6	26.8	1.1	25.2	26.0	26.8	27.4	28.5	30.9	0.9	30.1	30.1	30.5	31.1	32.2
SMI (kg/m^2^)	6.2	0.5	5.7	5.8	6.1	6.4	6.9	6.8	0.6	5.8	6.4	6.9	7.3	7.8	7.3	0.5	6.4	7.0	7.4	7.6	8.0	7.8	1.0	6.3	7.8	8.1	8.6	8.6
ECW/TBW	0.391	0.004	0.386	0.387	0.390	0.395	0.397	0.393	0.004	0.387	0.391	0.394	0.397	0.399	0.393	0.004	0.385	0.389	0.394	0.396	0.398	0.393	0.004	0.389	0.389	0.394	0.396	0.398
PhA (°)	4.7	0.4	4.1	4.4	4.8	5.0	5.1	4.6	0.4	3.9	4.3	4.6	4.9	5.3	4.8	0.5	4.1	4.4	4.7	5.2	5.5	4.7	0.4	4.3	4.3	4.7	4.7	5.3
Age (90–99 years)	N = 0							N = 4							N = 2							N = 1						
Age (years)	N/A	N/A	N/A	N/A	N/A	N/A	N/A	91.0	1.2	90.0	90.0	90.5	91.5	92.7	91.5	0.5	91.1	91.3	91.5	91.8	92.0	91.0	N/A	91.0	91.0	91.0	91.0	91.0
Height (cm)	N/A	N/A	N/A	N/A	N/A	N/A	N/A	162.6	8.3	151.5	160.5	164.7	166.7	170.9	157.0	0.1	156.8	156.9	157.0	157.0	157.1	162.3	N/A	162.3	162.3	162.3	162.3	162.3
Weight (kg)	N/A	N/A	N/A	N/A	N/A	N/A	N/A	55.9	6.2	48.4	53.0	55.9	58.8	63.5	65.8	3.7	62.5	63.9	65.8	67.6	69.0	79.6	N/A	79.6	79.6	79.6	79.6	79.6
BMI (kg/m^2^)	N/A	N/A	N/A	N/A	N/A	N/A	N/A	21.2	1.8	19.0	20.4	21.1	21.9	23.4	26.7	1.5	25.4	26.0	26.7	27.5	28.1	30.2	N/A	30.2	30.2	30.2	30.2	30.2
SMI (kg/m^2^)	N/A	N/A	N/A	N/A	N/A	N/A	N/A	7.2	1.4	5.8	6.2	6.8	7.7	9.0	3.8	3.8	0.4	1.9	3.8	5.7	7.2	8.7	N/A	8.7	8.7	8.7	8.7	8.7
ECW/TBW	N/A	N/A	N/A	N/A	N/A	N/A	N/A	0.393	0.003	0.389	0.391	0.393	0.394	0.397	0.396	0.002	0.394	0.395	0.396	0.397	0.398	0.398	N/A	0.398	0.398	0.398	0.398	0.398
PhA (°)	N/A	N/A	N/A	N/A	N/A	N/A	N/A	5.8	2.1	4.3	4.5	4.8	6.0	8.6	4.9	0.2	4.7	4.8	4.9	4.9	5.0	5.1	N/A	5.1	5.1	5.1	5.1	5.1

**Table 5:** Results of the datasets stratified by age and BMI in women SD: standard deviation; BMI: body mass index; SMI: skeletal muscle index; ECW/TBW: extracellular water/total body water; PhA: phase angle; N/A: insufficient sample size for analysis Percentile values were calculated from the distribution of observations within each age- and BMI-specific stratum

	BMI <18.5kg/m^2^ (N = 131)							BMI 18.5-24.9kg/m^2^ (N = 915)							BMI 25.0-29.9kg/m^2^ (N = 425)							BMI 30.0-39.9kg/m^2^ (N = 132)						
	Mean	SD	Percentile					Mean	SD	Percentile					Mean	SD	Percentile					Mean	SD	Percentile				
Age (10-19 years)	N = 1		5th	25th	50th	75th	95th	N = 14		5th	25th	50th	75th	95th	N = 4		5th	25th	50th	75th	95th	N = 0		5th	25th	50th	75th	95th
Age (years)	16.6	N/A	16.6	16.6	16.6	16.6	16.6	17.3	1.8	14.3	16.5	17.8	19.0	19.0	16.2	3.7	10.9	15.1	17.9	19.0	19.0	N/A	N/A	N/A	N/A	N/A	N/A	N/A
Height (cm)	158.0	N/A	158.0	158.0	158.0	158.0	158.0	163.1	8.2	152.4	158.2	161.0	168.3	175.9	158.7	7.7	148.8	155.3	159.5	162.9	167.5	N/A	N/A	N/A	N/A	N/A	N/A	N/A
Weight (kg)	44.6	N/A	44.6	44.6	44.6	44.6	44.6	56.1	8.1	44.2	51.0	54.1	59.9	70.2	65.6	6.9	56.2	63.6	67.4	69.4	72.5	N/A	N/A	N/A	N/A	N/A	N/A	N/A
BMI (kg/m^2^)	17.9	N/A	17.9	17.9	17.9	17.9	17.9	21.0	1.6	18.6	20.2	21.0	21.6	24.0	26.0	0.6	25.2	25.6	26.1	26.4	26.6	N/A	N/A	N/A	N/A	N/A	N/A	N/A
SMI (kg/m^2^)	6.2	N/A	6.2	6.2	6.2	6.2	6.2	6.7	0.7	5.9	6.3	6.4	7.2	7.9	6.4	0.6	5.6	6.0	6.5	6.9	6.9	N/A	N/A	N/A	N/A	N/A	N/A	N/A
ECW/TBW	0.371	N/A	0.371	0.371	0.371	0.371	0.371	0.374	0.006	0.367	0.370	0.372	0.379	0.385	0.381	0.003	0.377	0.379	0.381	0.383	0.384	N/A	N/A	N/A	N/A	N/A	N/A	N/A
PhA (°)	6.5	N/A	6.5	6.5	6.5	6.5	6.5	5.9	0.7	4.9	5.6	6.1	6.5	6.7	5.2	0.4	4.8	4.8	5.1	5.4	5.6	N/A	N/A	N/A	N/A	N/A	N/A	N/A
Age (20-29 years)	N = 5							N = 29							N = 2							N = 2						
Age (years)	21.0	2.0	20.0	20.0	20.0	20.0	24.0	24.7	3.0	20.4	22.0	24.0	28.0	29.0	23.0	2.0	21.2	22.0	23.0	24.0	24.8	25.0	2.0	23.2	24.0	25.0	26.0	26.8
Height (cm)	163.0	4.5	156.0	163.0	165.5	166.0	166.1	160.4	7.0	150.7	156.5	159.5	164.0	173.0	172.5	1.6	171.1	171.7	172.5	173.3	173.9	156.5	3.5	153.3	154.7	156.5	158.2	159.6
Weight (kg)	47.6	2.0	44.6	48.0	48.2	48.6	49.2	53.4	6.2	44.9	48.3	52.9	56.6	64.3	75.1	0.9	74.3	74.7	75.1	75.6	75.9	83.7	10.3	74.4	78.6	83.7	88.9	93.0
BMI (kg/m^2^)	17.9	0.4	17.4	17.6	17.9	18.3	18.4	20.7	1.2	19.2	19.9	20.4	21.3	22.4	25.3	0.1	25.1	25.2	25.3	25.3	25.4	34.1	2.7	31.7	32.8	34.1	35.5	36.5
SMI (kg/m^2^)	5.9	0.2	5.6	5.7	5.8	6.1	6.2	6.3	0.6	5.4	5.8	6.3	6.8	7.3	7.2	0.7	6.6	6.9	7.2	7.6	7.8	8.2	0.3	7.9	8.0	8.2	8.3	8.5
ECW/TBW	0.386	0.009	0.371	0.385	0.391	0.393	0.393	0.378	0.005	0.370	0.374	0.379	0.382	0.386	0.373	0.008	0.366	0.369	0.373	0.377	0.380	0.380	0.007	0.374	0.376	0.380	0.383	0.385
PhA (°)	4.7	0.6	4.1	4.2	4.5	4.7	5.7	5.5	0.5	4.7	5.1	5.4	5.8	6.3	5.9	0.7	5.3	5.6	5.9	6.3	6.5	5.7	0.3	5.4	5.6	5.7	5.9	6.0
Age (30-39 years)	N = 11							N = 26							N = 5							N = 5						
Age (years)	35.3	3.4	30.0	32.0	36.0	38.5	39.0	35.0	3.1	30.0	32.3	35.5	38.0	39.0	35.2	2.0	33.2	34.0	35.0	35.0	38.2	36.4	2.1	33.6	36.0	36.0	38.0	38.8
Height (cm)	159.4	4.2	152.4	157.5	160.0	161.7	165.5	158.9	4.7	153.4	155.6	157.8	161.0	168.0	159.5	5.8	150.7	161.6	161.9	162.8	163.3	158.9	2.0	156.2	157.0	160.0	160.6	160.9
Weight (kg)	44.1	3.2	39.8	42.0	43.1	46.4	48.7	53.8	4.6	47.5	50.1	54.5	58.4	59.3	70.0	5.3	62.1	69.6	70.8	74.1	75.1	84.2	7.1	75.0	78.9	86.7	86.7	93.0
BMI (kg/m^2^)	17.3	0.8	16.1	16.7	17.6	18.0	18.4	21.3	1.7	19.0	19.9	20.9	22.5	24.5	27.5	0.8	26.5	26.7	27.5	28.3	28.4	33.3	2.9	30.4	30.4	33.6	33.9	37.5
SMI (kg/m^2^)	5.5	0.5	4.9	5.1	5.5	5.7	6.5	6.2	0.4	5.7	6.0	6.3	6.4	6.7	7.0	0.4	6.4	6.9	7.1	7.2	7.4	7.6	0.6	6.8	7.5	7.6	7.9	8.2
ECW/TBW	0.387	0.005	0.380	0.385	0.387	0.391	0.395	0.384	0.005	0.377	0.381	0.385	0.387	0.390	0.381	0.002	0.378	0.380	0.382	0.383	0.384	0.381	0.006	0.375	0.378	0.379	0.384	0.390
PhA (°)	4.7	0.4	4.1	4.4	4.7	5.2	5.2	5.1	0.4	4.5	4.8	5.1	5.5	5.9	5.4	0.2	5.2	5.4	5.5	5.5	5.6	5.6	0.4	4.9	5.5	5.8	5.9	5.9
Age (40-49 years)	N = 6							N = 50							N = 28							N = 20						
Age (years)	45.3	3.9	41.2	44.0	48.0	50.0	51.8	45.2	2.4	41.0	43.3	45.0	47.0	48.6	44.5	2.7	41.0	42.8	44.0	47.0	49.0	44.6	2.7	40.0	42.8	45.0	47.0	48.1
Height (cm)	159.6	2.5	156.7	158.5	161.0	161.0	163.8	158.5	5.9	149.0	155.5	157.9	161.0	169.8	159.8	5.6	150.8	155.8	160.0	162.6	169.6	158.7	5.7	150.8	154.5	158.2	162.0	169.2
Weight (kg)	44.2	2.1	42.1	42.9	44.3	45.8	47.8	53.0	5.2	45.6	48.9	52.9	56.5	61.1	70.0	6.6	60.6	65.3	70.0	74.5	82.1	82.8	7.9	71.6	76.5	81.3	90.1	95.1
BMI (kg/m^2^)	17.4	0.5	16.7	17.1	17.2	17.7	18.2	21.1	1.7	18.7	19.7	21.0	22.5	23.9	27.4	1.4	25.5	26.1	27.0	28.4	29.7	32.9	2.4	30.5	31.3	32.0	33.3	36.7
SMI (kg/m^2^)	5.6	0.5	5.0	5.4	5.5	5.8	6.4	6.0	0.5	5.4	5.8	6.0	6.3	6.8	6.9	0.5	6.1	6.6	6.9	7.3	7.7	7.6	0.5	7.0	7.3	7.7	7.9	8.3
ECW/TBW	0.389	0.004	0.381	0.385	0.387	0.391	0.392	0.386	0.005	0.378	0.382	0.386	0.389	0.394	0.385	0.005	0.377	0.381	0.384	0.389	0.392	0.385	0.005	0.377	0.382	0.385	0.388	0.391
PhA (°)	4.7	0.3	4.5	4.7	4.7	4.8	5.3	4.9	0.5	4.0	4.6	5.0	5.2	5.6	5.2	0.5	4.5	4.8	5.2	5.6	6.0	5.3	0.3	4.9	5.1	5.3	5.6	5.9
Age (50-59 years)	N = 12							N = 77							N = 52							N = 41						
Age (years)	55.1	2.9	50.0	53.8	55.5	57.0	59.0	54.5	2.7	50.0	52.0	55.0	57.0	58.2	55.1	2.7	51.0	53.0	55.0	57.3	59.0	55.2	2.7	52.0	52.0	55.0	57.0	59.0
Height (cm)	159.3	4.0	153.6	155.9	160.0	163.1	164.4	158.5	5.9	148.0	154.5	158.7	161.1	169.1	158.1	4.2	151.0	155.3	157.4	161.0	165.6	154.8	5.0	148.3	151.4	155.4	158.4	160.9
Weight (kg)	44.0	3.4	38.4	42.1	43.7	46.3	48.9	54.5	5.4	45.0	51.1	55.5	57.9	63.0	68.7	5.9	57.5	64.9	69.4	72.7	77.9	79.3	7.5	68.5	73.1	78.7	85.2	93.0
BMI (kg/m^2^)	17.3	1.0	15.8	16.6	17.5	18.2	18.4	21.7	1.8	18.9	20.1	21.8	23.3	24.4	27.4	1.6	25.1	26.1	27.6	28.5	29.8	33.1	2.2	30.1	31.3	32.5	34.5	36.8
SMI (kg/m^2^)	5.6	0.5	5.0	5.3	5.6	5.7	6.3	6.0	0.5	5.1	5.7	6.0	6.4	7.0	6.8	0.5	6.1	6.5	6.8	7.1	7.6	7.4	0.5	6.6	7.1	7.4	7.7	8.2
ECW/TBW	0.388	0.006	0.380	0.383	0.389	0.391	0.396	0.386	0.005	0.378	0.383	0.386	0.389	0.395	0.386	0.006	0.376	0.382	0.386	0.390	0.396	0.386	0.005	0.379	0.383	0.386	0.389	0.394
PhA (°)	4.7	0.4	4.2	4.5	4.8	4.9	5.3	4.9	0.5	4.1	4.6	5.0	5.3	5.7	5.1	0.5	4.5	4.8	5.1	5.4	5.9	5.1	0.4	4.6	4.9	5.1	5.3	5.7
Age (60-69 years)	N = 29							N = 189							N = 116							N = 39						
Age (years)	65.5	2.6	60.4	64.0	66.0	67.0	69.0	64.8	2.9	60.0	63.0	65.0	68.0	69.0	65.3	2.8	60.0	63.8	66.0	68.0	69.0	64.6	2.9	60.0	62.5	64.0	67.0	69.0
Height (cm)	155.3	6.2	145.2	151.7	155.0	159.0	166.5	155.0	5.6	145.9	151.7	155.5	158.5	163.6	154.2	5.4	144.3	151.0	154.3	158.1	161.6	152.9	5.1	144.3	149.8	152.8	156.9	160.2
Weight (kg)	41.9	4.0	35.2	40.0	42.1	44.4	48.4	53.2	5.6	43.5	49.3	53.2	57.1	63.1	64.8	5.3	55.9	61.1	64.5	68.7	73.3	76.4	8.1	65.9	70.9	76.3	80.0	90.4
BMI (kg/m^2^)	17.4	0.9	15.5	17.2	17.7	17.9	18.3	22.1	1.8	18.9	20.8	22.2	23.6	24.7	27.2	1.4	25.3	26.0	27.2	28.3	29.8	32.6	2.3	30.2	31.0	31.8	33.3	37.6
SMI (kg/m^2^)	5.1	0.5	4.2	4.9	5.3	5.4	5.8	5.9	0.5	5.2	5.5	5.9	6.3	6.6	6.6	0.5	5.8	6.3	6.6	6.9	7.4	7.1	0.5	6.4	6.7	7.1	7.5	8.0
ECW/TBW	0.390	0.005	0.383	0.387	0.391	0.394	0.398	0.388	0.005	0.379	0.385	0.389	0.392	0.396	0.388	0.006	0.379	0.384	0.388	0.393	0.398	0.390	0.004	0.384	0.388	0.390	0.394	0.396
PhA (°)	4.3	0.4	3.6	4.0	4.3	4.6	4.9	4.7	0.5	4.0	4.4	4.7	5.0	5.5	4.9	0.5	4.2	4.5	4.9	5.2	5.7	4.9	0.4	4.3	4.6	5.0	5.2	5.5
Age (70-79 years)	N = 45							N = 380							N = 168							N = 21						
Age (years)	74.6	2.7	70.2	72.0	74.0	77.0	79.0	75.0	2.6	71.0	73.0	75.0	77.0	79.0	74.3	2.7	70.0	72.0	74.0	76.0	79.0	74.3	2.2	71.0	73.0	74.0	75.0	77.0
Height (cm)	149.6	5.4	139.4	147.0	150.5	153.2	157.0	151.3	5.2	142.0	148.0	151.0	154.8	159.6	150.1	5.5	140.9	146.4	150.1	154.0	158.4	148.5	6.5	137.9	146.5	149.0	153.0	157.0
Weight (kg)	39.2	3.7	32.5	36.5	39.7	42.0	43.6	50.8	5.3	42.2	47.0	50.9	53.9	59.1	60.8	5.0	52.4	57.2	60.9	64.2	69.2	71.7	8.0	58.9	67.9	71.6	76.7	85.0
BMI (kg/m^2^)	17.5	0.9	15.3	17.1	17.8	18.2	18.4	22.2	1.8	19.3	20.7	22.3	23.6	24.7	27.0	1.4	25.2	25.9	26.6	28.1	29.5	32.4	2.2	30.1	30.9	31.2	33.8	36.5
SMI (kg/m^2^)	5.0	0.5	4.2	4.8	5.1	5.3	5.6	5.8	0.5	5.0	5.5	5.7	6.1	6.6	6.3	0.5	5.5	6.0	6.4	6.6	7.1	6.9	0.5	6.3	6.6	7.0	7.2	7.4
ECW/TBW	0.392	0.005	0.384	0.390	0.393	0.396	0.399	0.392	0.005	0.384	0.389	0.392	0.396	0.399	0.392	0.005	0.385	0.389	0.393	0.396	0.399	0.394	0.004	0.389	0.392	0.394	0.397	0.398
PhA (°)	4.3	0.4	3.8	4.0	4.2	4.5	5.1	4.5	0.4	3.8	4.2	4.5	4.7	5.2	4.6	0.4	4.0	4.3	4.5	4.9	5.3	4.6	0.3	4.3	4.5	4.6	4.7	5.2
Age (80-89 years)	N = 22							N = 148							N = 47							N = 4						
Age (years)	82.0	1.5	80.0	81.0	82.0	83.0	84.0	82.4	2.2	80.0	81.0	82.0	84.0	86.7	82.6	2.1	80.0	81.0	82.0	84.0	86.7	81.3	1.3	80.0	80.0	81.0	82.3	82.9
Height (cm)	148.8	4.8	141.9	146.1	148.5	152.4	157.0	148.9	5.2	141.1	146.0	149.2	152.4	157.3	148.7	5.2	140.7	145.3	148.0	151.2	157.0	144.7	2.6	141.9	142.5	144.4	146.6	148.0
Weight (kg)	37.6	2.7	34.2	35.4	37.7	39.3	42.2	48.6	5.2	40.7	45.2	48.8	52.4	56.4	58.7	4.7	50.6	55.4	58.4	63.3	65.6	69.5	2.8	66.3	67.7	69.1	70.9	73.1
BMI (kg/m^2^)	17.0	1.1	14.7	16.4	17.3	17.9	18.2	21.9	1.8	18.9	20.5	21.9	23.6	24.6	26.6	1.4	25.0	25.4	26.3	27.1	29.6	33.2	1.3	31.4	32.8	33.7	34.1	34.3
SMI (kg/m^2^)	4.9	0.4	4.0	4.7	4.9	5.2	5.3	5.5	0.5	4.5	5.2	5.5	5.8	6.3	6.2	0.5	5.5	5.8	6.3	6.6	6.9	6.9	0.3	6.6	6.7	6.9	7.1	7.3
ECW/TBW	0.394	0.004	0.386	0.391	0.396	0.398	0.399	0.394	0.004	0.388	0.392	0.395	0.397	0.399	0.394	0.004	0.387	0.392	0.395	0.398	0.399	0.390	0.006	0.382	0.388	0.392	0.394	0.396
PhA (°)	4.1	0.4	3.6	3.9	4.1	4.4	4.9	4.3	0.3	3.8	4.1	4.3	4.5	4.9	4.5	0.4	3.9	4.3	4.5	4.7	5.1	4.8	0.5	4.3	4.5	4.7	5.0	5.6
Age (90-99 years)	N = 0							N = 2							N = 3							N = 0						
Age (years)	N/A	N/A	N/A	N/A	N/A	N/A	N/A	94.0	3.0	91.3	92.5	94.0	95.5	96.7	92.7	2.5	90.2	91.0	92.0	94.0	95.6	N/A	N/A	N/A	N/A	N/A	N/A	N/A
Height (cm)	N/A	N/A	N/A	N/A	N/A	N/A	N/A	141.5	7.5	134.8	137.8	141.5	145.3	148.3	148.7	2.8	145.5	147.1	149.1	150.5	151.5	N/A	N/A	N/A	N/A	N/A	N/A	N/A
Weight (kg)	N/A	N/A	N/A	N/A	N/A	N/A	N/A	43.8	0.5	43.3	43.5	43.8	44.0	44.2	57.2	3.9	53.5	54.6	56.0	59.2	61.8	N/A	N/A	N/A	N/A	N/A	N/A	N/A
BMI (kg/m^2^)	N/A	N/A	N/A	N/A	N/A	N/A	N/A	22.1	2.6	19.8	20.8	22.1	23.4	24.4	25.9	0.9	25.2	25.3	25.3	26.2	26.9	N/A	N/A	N/A	N/A	N/A	N/A	N/A
SMI (kg/m^2^)	N/A	N/A	N/A	N/A	N/A	N/A	N/A	5.5	0.8	4.8	5.1	5.5	5.9	6.2	6.0	0.3	5.7	5.9	6.1	6.3	6.4	N/A	N/A	N/A	N/A	N/A	N/A	N/A
ECW/TBW	N/A	N/A	N/A	N/A	N/A	N/A	N/A	0.396	0.003	0.393	0.395	0.396	0.398	0.399	0.393	0.004	0.389	0.390	0.391	0.395	0.398	N/A	N/A	N/A	N/A	N/A	N/A	N/A
PhA (°)	N/A	N/A	N/A	N/A	N/A	N/A	N/A	4.2	0.5	3.8	4.0	4.2	4.5	4.7	4.6	0.1	4.5	4.5	4.5	4.6	4.7	N/A	N/A	N/A	N/A	N/A	N/A	N/A

## Discussion

The mean PhA values measured using the InBody770 were consistently lower across all strata than those reported in the German database [4]. Because this survey confirmed normal fluid balance using ECW/TBW and verified that the measurements were properly performed based on impedance, gross measurement errors are unlikely to explain the observed differences. Previous studies have reported population-related differences in PhA [4,5,7]. However, population characteristics alone may not fully explain the observed differences. Differences in impedance analyzers, calibration procedures, measurement methodologies, and participant selection criteria between studies may also have contributed to the observed discrepancies. Therefore, the lower PhA values observed in this survey compared with the German reference data cannot be attributed solely to population characteristics.

Currently, reports providing PhA reference values stratified by age, sex, and BMI are limited [4]. To our knowledge, this is the first survey to report such values specifically for the Japanese population. In calculating SPA for conditions in which diagnostic criteria differ among populations, PhA reference values should account for population differences. Therefore, the PhA reference values measured using the InBody770 in Japanese individuals may serve as valuable data for calculating SPA in Japanese and other Asian populations. The use of SPA enables comparisons of PhA-related findings across different clinical settings and studies and may contribute to advancing research on PhA in various fields.

However, this survey was conducted at a single medical facility. Therefore, potential selection bias should be considered when evaluating the generalizability of the findings derived from this survey. Additionally, BIA measurements in this survey were performed using a single device, the InBody770 body composition analyzer. Because compatibility among different BIA devices is limited, reference values should ideally be established using measurements obtained from a single device under standardized conditions [28].

Furthermore, this survey provided PhA reference values measured using the InBody770 for Japanese individuals with normal fluid balance (ECW/TBW <0.4). SPA calculation requires the mean and standard deviation of PhA values obtained from a “healthy population.” However, it is difficult to evaluate whether participants meet the widely adopted WHO definition of health or establish a baseline health level in a target population. Previously, the absence of clinical conditions affecting fluid balance was assessed using questionnaires. In contrast, this survey used the ECW/TBW ratio as an objective indicator of fluid balance. If impedance measurements confirm that InBody assessments have been properly conducted, ECW/TBW may provide a practical criterion for SPA calculations. However, ECW/TBW should be interpreted as an indicator of fluid balance rather than a surrogate marker of overall health status.

Finally, because PhA is influenced by age, sex, and BMI, SPA may enable more meaningful comparisons across different studies and clinical settings. The age-, sex-, and BMI-specific reference values presented in this survey may serve as valuable data for the calculation of SPA in Japanese and other Asian populations.

This survey has several limitations. First, a substantial proportion of the original datasets was excluded, primarily because duplicate measurements were removed and individuals with an ECW/TBW ratio ≥0.4 were excluded. Therefore, selection bias cannot be ruled out, and the final analytical sample may not fully represent the original study population. Second, there was variability in the number of samples across the stratified subgroups. Interpretation of the results should therefore take the sample size of each subgroup into account. In particular, subgroups with ≤17 samples should be interpreted cautiously, considering that the minimum sample size reported in a previous study presenting sex- and age-specific PhA reference values was 17 [5]. Third, the normality of the PhA distribution was not observed in some subgroups. In general, the use of mean values and standard deviations for PhA assumes that the data are normally distributed. Previous studies did not mention the normality of the PhA distributions; however, percentile values were presented alongside means and standard deviations [4,5]. Similarly, the present survey provided mean values, standard deviations, and percentile values. Nevertheless, if these PhA reference values are to be applied for SPA calculation, securing sufficient sample sizes and validating distributional assumptions in all subgroups remains an important issue for future research. Fourth, the recommended conditions for InBody measurements, such as fasting status and morning measurements, could not be fully controlled. Variations in hydration status, recent food intake, physical activity, and measurement timing may influence impedance measurements and consequently affect PhA values. Although participants were instructed to follow standardized measurement procedures, residual variability related to these factors may have contributed to measurement variability in the present survey. Fifth, because the survey period extended from 2010 to 2025, the potential effects of long-term changes in device maintenance procedures, calibration status, or software versions could not be fully evaluated. Sixth, the study population consisted of individuals attending a medical institution. Therefore, caution is required when generalizing these findings to the broader Japanese population. Seventh, although SPA reference values should ideally be derived from a “healthy population,” it was difficult to apply the WHO definition of health in this survey. Therefore, we referred to previous criteria excluding individuals with clinical conditions that may affect fluid balance, such as renal, endocrine, or cardiac diseases, and instead used ECW/TBW as an objective indicator of fluid status [4]. Finally, although the results of this survey indicate normal fluid balance based on ECW/TBW, diagnostic information was not available for all participants. In addition, because this was a retrospective survey, the validity of recorded diagnoses and disease severity could not be verified, and medication data were unavailable. Therefore, the potential effects of specific diseases and medications on fluid balance and PhA could not be directly evaluated.

## Conclusions

This survey established age-, sex-, and BMI-specific PhA reference values measured using the InBody770 in Japanese individuals with normal fluid balance, defined as an ECW/TBW ratio <0.4. The results demonstrated lower PhA values across all strata compared with previously reported German reference values. These findings provide reference data for calculating SPA in Japanese individuals and may support future research and clinical applications of PhA in Asian populations. However, because the study population consisted of individuals attending a single medical institution and normal fluid balance does not necessarily indicate overall health, further validation in larger and more representative populations is warranted.
